# The first complete chloroplast genome of *Cymodocea rotundata* Asch. & Schweinf. 1870 (Cymodoceaceae), an Indo-Pacific seagrass

**DOI:** 10.1080/23802359.2024.2432370

**Published:** 2024-11-26

**Authors:** Mingzhong Liu, Jiaxin Wu, Yunfeng Shi, Rongrong Shan

**Affiliations:** Key Laboratory for Coastal Marine Eco-Environment Process and Carbon Sink of Hainan province, Yazhou Bay Innovation Institute, College of Ecology and Environment, Hainan Tropical Ocean University, Sanya, China

**Keywords:** chloroplast genome, *Cymodocea rotundata*, phylogenetic analysis, seagrass

## Abstract

*Cymodocea rotundata* Asch. & Schweinf. 1870 (Cymodoceaceae) is a seagrass found in the tropical and temperate Indo-Pacific coastal waters. Seagrass beds composed of *C. rotundata* and other seagrasses form ecologically valuable ecosystems. In this study, the complete chloroplast genome of *C. rotundata* was sequenced and characterized for the first time. It is a circular genome of 158,311 bp in length, consisting of a large single-copy region (88,451 bp), a small single-copy region (18,836 bp), and a pair of inverted repeats (25,512 bp). A total of 130 genes were annotated, including 86 protein-coding genes (PCGs), 36 transfer RNA (tRNA) genes, and 8 ribosomal RNA (rRNA) genes. Seven PCGs and 6 tRNA genes contain 1 intron, and 3 PCGs contain 2 introns. In addition, 7 PCGs, 7 tRNA genes, and all the rRNA genes are multi-copy genes with 2 copies. Phylogenetic analysis shows that *C. rotundata* clusters with *Syringodium isoetifolium* in one clade, both belonging to the family Cymodoceaceae. This study provides new information to support further research on the phylogeny of Cymodoceaceae.

## Introduction

Seagrasses are marine flowering plants (Chanda 2022). *Cymodocea rotundata* Asch. & Schweinf. 1870 (Cymodoceaceae) is a seagrass of the family Cymodoceaceae (https://www.worldfloraonline.org/taxon/wfo-0000764885). *C. rotundata* has creeping and slender rhizomes. Nodes along the rhizomes each have 1–3 roots and 1 erect shoot. Each shoot produces 2–5 leaves. The leaves are 7–15 cm long and 4 mm wide with 9–15 veins. The leaf sheaths are 1.4–4 cm in length. The fruits are small and semicircular (https://www.worldfloraonline.org/taxon/wfo-0000764885). Flowering in *C. rotundata* is rare in natural seagrass beds (McMillan et al. 1982). *C. rotundata* is distributed across the tropical and temperate Indo-Pacific coastal waters (https://www.iucnredlist.org/species/173363/6999692). On Hainan Island (108°37′–111°03′E; 18°10′–20°10′N), China, the site of this study, *C. rotundata* is found in the eastern coastal waters, where it usually grows alongside *Thalassia hemprichii* and other seagrasses in the local seagrass beds.

A seagrass bed is a marine ecosystem that provides multiple ecological services, such as carbon sequestration and high primary productivity (Chanda 2022). It also plays an important role in sustainable seagrass-based fisheries (Nordlund et al. 2018). To date, 74 seagrass species have been recorded globally, classified into 6 families and 13 genera (Short et al. 2011). Among these species, *C. rotundata*, *C. nodosa*, *C. serrulate*, and *C. angustata* comprise the entire genus Cymodocea in the family Cymodoceaceae (Short et al. 2001), and no chloroplast genome sequences for any Cymodocea species are available. *C. rotundata* is efficient in removing nitrate and phosphate in water (Soumya et al. 2015), but the seagrass bed area containing this species is shrinking, owing to the marine aquaculture sewage as the main pollution source (Thomsen et al. 2020). The restoration and conservation of the seagrass bed ecosystem are a pressing global concern. To address this, the basic genetic knowledge of *C. rotundata* needs to be expanded. Therefore, we conducted the first sequencing of the *C. rotundata* chloroplast genome. The complete genome sequence was characterized, and its phylogenetic relationships with other seagrasses were elucidated for the first time, contributing to the future conservation and restoration of the seagrass ecosystem.

## Materials and methods

### Sampling and sample preservation

**Figure 1.**
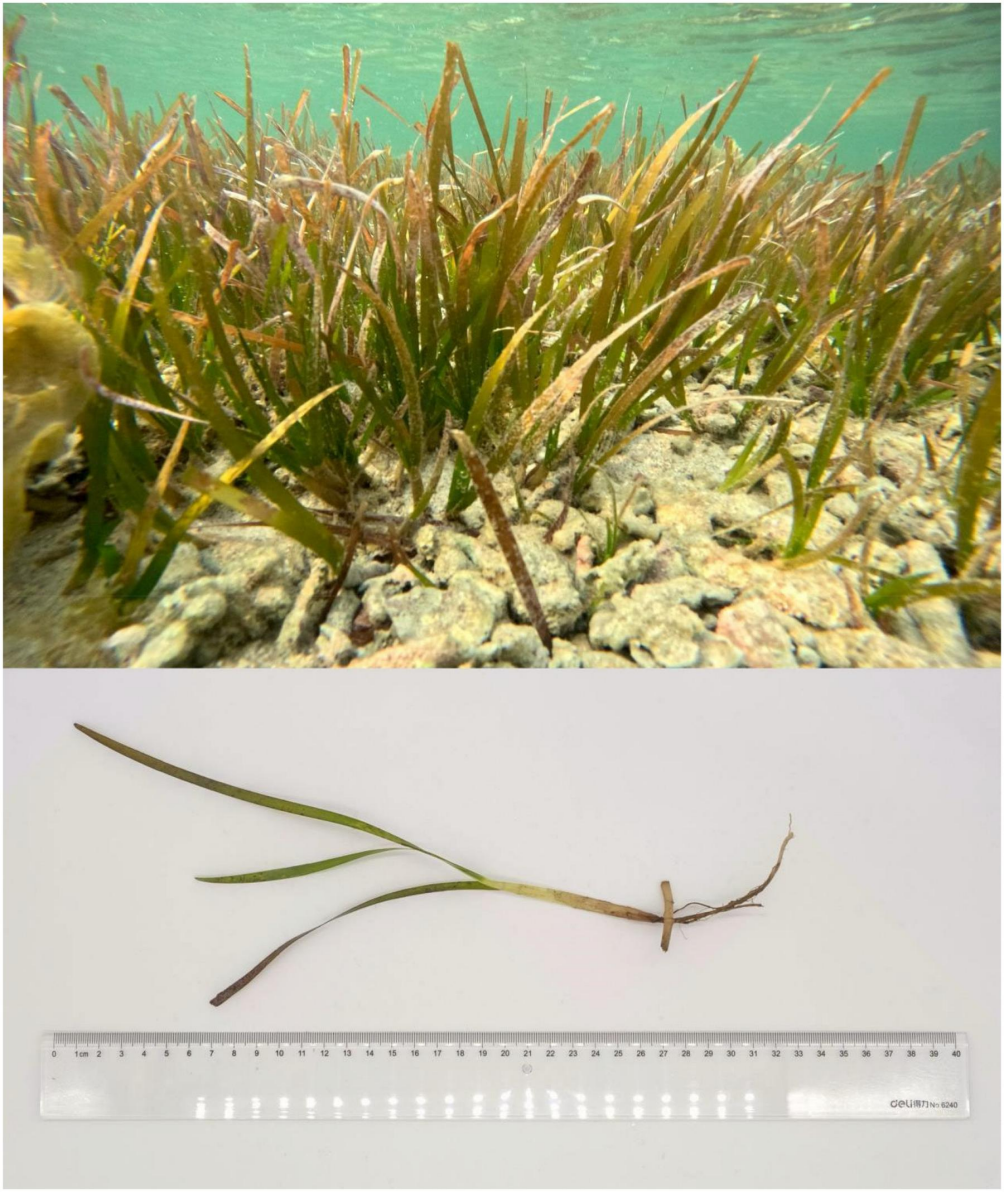
Photo of *Cymodocea rotundata* in a seagrass bed, taken by Mingzhong Liu in Xiaodonghai (18°12′51.3″N, 109°30′31.8″E), Hainan Island, China. *C. rotundata* is a marine herb with slender creeping rhizomes and 2–5 leaves on each shoot. Leaves are approximately 4 mm in width and 7–15 mm in length. Photo credit: Mingzhong Liu.

Xiaodonghai (18°12′51.3″N, 109°30′31.8″E) is a bay on the southern coast of Hainan Island, China. A total of 2.31 g of *C. rotundata* (Figure 1) fresh leaves were collected from this location. A plant voucher specimen (01R0011) was deposited at the High-tech Center Building NO.3, Shanghai Road, Qingdao, China (Contact: Jinhu Mu, mujh@biomarker.com.cn).

### DNA extraction, sequencing, assembly and annotation

The Cetyltrimethylammonium Bromide (CTAB) method (Sahu et al. 2012) was used to extract chloroplast genomic DNA from the fresh leaves for library construction. A sequencing library was generated using the VAHTS Universal DNA Library Prep Kit (ND607-02), and the library was sequenced on the Illumina Novaseq 6000 platform (Novogene, Sacramento, CA), with paired-end reads of 150 bp each. A total of 35,999,109 raw reads were generated. Sequencing depth detection was performed with Samtools v.1.7 (Li et al. 2009). The raw reads were quality filtered through fastp v0.20.0 (https://github.com/OpenGene/fastp) by removing the adapters and low-quality reads. After filtering, 35,279,127 clean reads were obtained, and the clean reads were assembled into the complete chloroplast genome using GetOrganelle v1.1.7 (Jin et al. 2020). The genome was annotated and manually adjusted with Geseq (Tillich et al. 2017; https://chlorobox.mpimp-golm.mpg.de/geseq.html). The annotation included Protein Coding Genes (PCGs), ribosomal RNA (rRNA), and transfer RNA (tRNA). The complete chloroplast genome sequence of *C. rotundata* was submitted to the GenBank database (https://www.ncbi.nlm.nih.gov/) under the accession number OQ735398. The chloroplast genome map was visualized with OGDraw (Greiner et al. 2019; https://chlorobox.mpimp-golm.mpg.de/OGDraw.html).

### Phylogenetic analysis

The complete chloroplast genome sequences of 28 related species, including *Gossypium hirsutum* as the outgroup, were selected from the GenBank database for phylogenetic analysis. Chloroplast genome sequences of all 29 species were aligned with MAFFT v7.5 (Katoh and Standley 2013). The phylogenetic relationships of these species were constructed based on the complete chloroplast sequences using Bayesian inference (BI) analysis. The BI tree was generated using MrBayes 3.2.6 (Ronquist and Huelsenbeck 2003), with GTR+F + I + G4 identified as the best-fit evolutionary model by ModelFinder (Kalyaanamoorthy et al. 2017).

## Results

**Figure 2.**
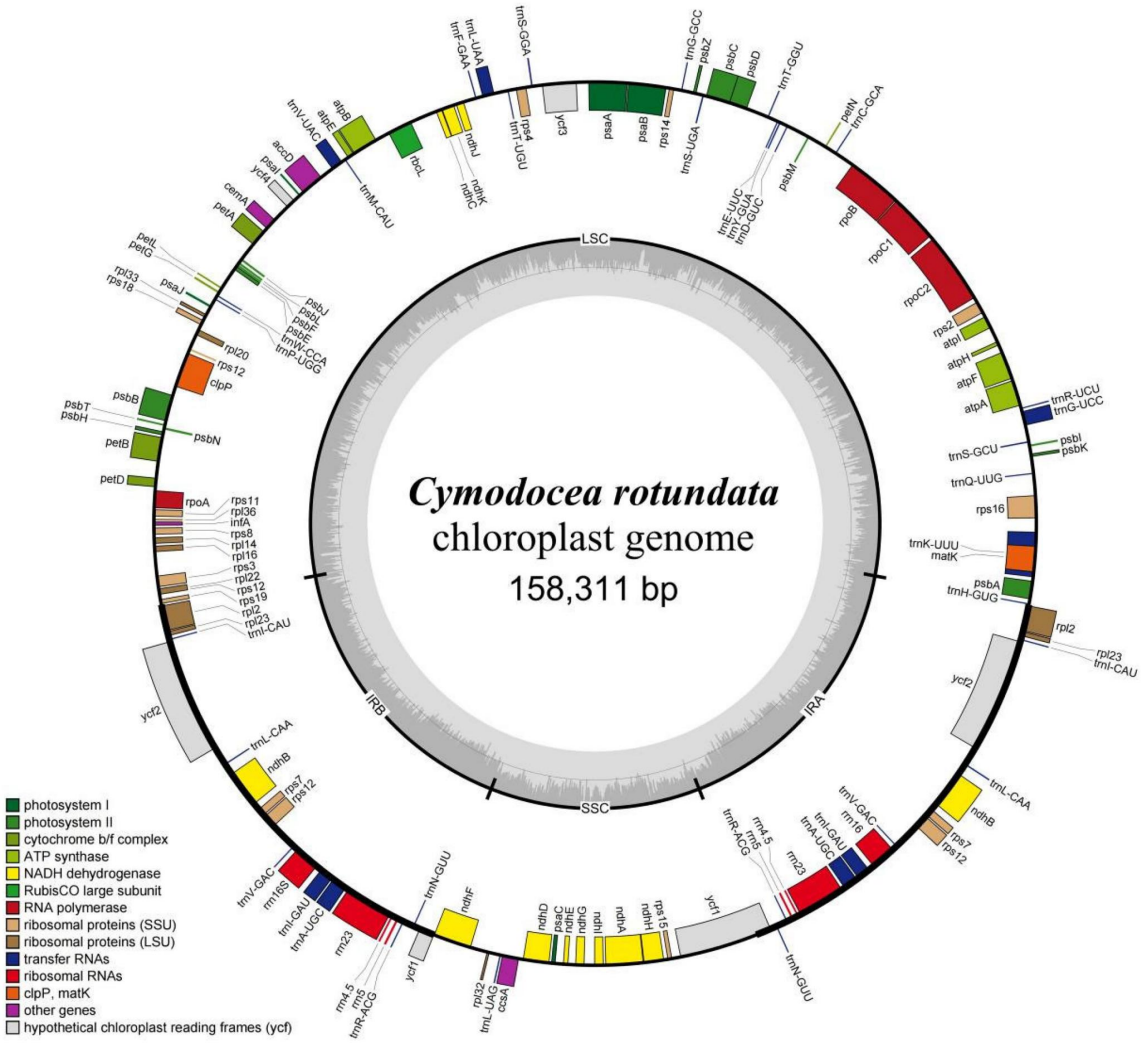
Chloroplast genome map of *Cymodocea rotundata* visualized through OGDRAW. The genes inside the outer circle represent the clockwise direction of transcription, and the genes outside this circle represent the counterclockwise direction. Genes in different functional groups are labeled in various colors. Marks on the inner circle delineate the Large Single-Copy, Small Single-Copy, and inverted repeats regions. The dark grey and light grey zones inside this circle correspond to GC and AT content, respectively, with the grey circle representing the 50% threshold.

The chloroplast genome of *C. rotundata* is 158,311 bp in length, with an average sequencing depth of 2753.81× and the minimum sequencing depth of 112× (supplemental Figure S1). The genome is circular (Figure 2). The genome contains 88,451 bp in the Large Single-Copy region as the longest region (GC%: 34.1), 18,836 bp in the Small Single-Copy region (GC%: 29.2), and 25,512 bp each in a pair of inverted repeats (GC%: 42.7). The overall GC content of the chloroplast genome is 36.32%. There are 130 genes annotated in total, including 86 protein-coding genes (PCGs), 36 transfer-RNA (tRNA) genes, and 8 ribosomal-RNA (rRNA) genes. Among these genes, there are 7 PCGs (*ndh*A, *ndh*B, *pet*B, *atp*F, *rpl*2, *rps*16, *rpo*C1) and 6 tRNA genes (*trn*A-UGC, *trn*G-UCC, *trn*I-GAU, *trn*K-UUU, *trn*L-UAA, *trn*V-UAC) that contain 1 intron, while 3 PCGs (*rps*12, *clp*P, *ycf*3) contain 2 introns. Finally, 7 of the PCGs (*ndh*B, *rpl*2, *rpl*23, *rps*12, *rps*7, *ycf*1, *ycf*2), 7 of the tRNA genes (*trn*A-UGC, *trn*I-CAU, *trn*L-CAA, *trn*N-GUU, *trn*R-ACG, *trn*V-GAC, *trn*I-GAU), and all of the rRNA genes are multi-copy genes with 2 copies. All the tRNA genes have a cloverleaf secondary structure. The structures of the 11 protein-coding cis-splicing genes (*rps16*, *atpF*, *rpoC1*, *ycf3*, *clpP*, *petB*, *rpl2*, *ndhB*, *ndhA*, *ndhB*, *rpl2*) and 1 trans-splicing gene (*rps12*) are presented in supplemental Figures S2 and S3, respectively.

**Figure 3.**
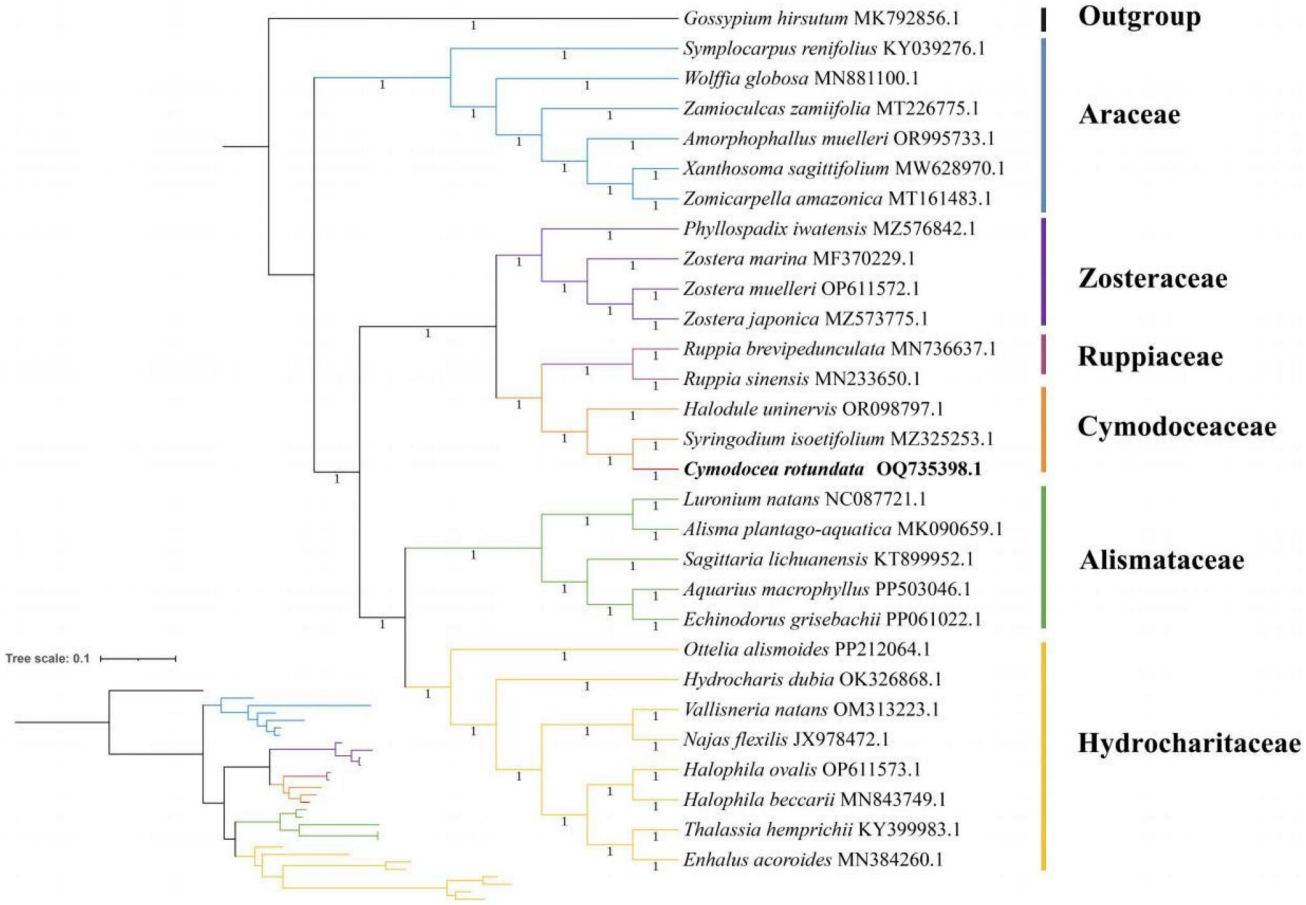
Bayesian inference analysis (BI Tree) of *Cymodocea rotundata* and 28 other species based on complete chloroplast genome sequences. The numbers represents the posterior probabilities of the BI model. *Gossypium hirsutum* MK792856.1 (Zhou et al. 2022) is the outgroup. *C. rotundata* OQ735398 is in bold. The following sequences were included in the analysis: *Enhalus acoroides* MN384260.1, *Thalassia hemprichii* KY399983.1, *Halophila beccarii* MN843749.1 (Yu et al. 2020c), *Halophila ovalis* OP611573.1 (Chen et al. 2021), *Najas flexilis* JX978472.1 (Peredo et al. 2013), *Vallisneria natans* OM313223.1, *Hydrocharis dubia* OK326868.1, *Ottelia alismoides* PP212064.1, *Echinodorus grisebachii* PP061022.1, *Aquarius macrophyllus* PP503046.1, *Sagittaria lichuanensis* KT899952.1 (Luo et al. 2016), *Alisma plantago-aquatica* MK090659.1 (Liang et al. 2019), *Luronium natans* NC087721.1, *Syringodium isoetifolium* MZ325253.1 (Ruan et al. 2022), *Halodule uninervis* OR098797.1 (Liu et al. 2024), *Ruppia sinensis* MN233650.1 (Yu et al. 2019), *Ruppia brevipedunculata* MN736637.1 (Yu et al. 2020b), *Zostera japonica* MZ573775.1 (Chen et al. 2021), *Zostera muelleri* OP611572.1, *Zostera marina* MF370229.1 (Xing and Guo 2018), *Phyllospadix iwatensis* MZ576842.1 (Chen et al. 2021), *Zomicarpella amazonica* MT161483.1 (Abdullah et al. 2020), *Xanthosoma sagittifolium* MW628970.1), *Amorphophallus muelleri* OR995733.1, *Zamioculcas zamiifolia* MT226775.1 (Abdullah et al. 2020), *Wolffia globosa* MN881100.1 (Park et al. 2020), *Symplocarpus renifolius* KY039276.1 (Choi et al. 2017), *Gossypium hirsutum* MK792856.1 (Zhou et al. 2022).

Complete chloroplast genome sequences of 28 additional species were selected for phylogenetic analysis with *C. rotundata* (Figure 3). Apart from *Gossypium hirsutum* in the family Malvaceae as the outgroup, the remaining 27 species belong to the families Hydrocharitaceae, Alismataceae, Cymodoceaceae, Ruppiaceae, Zosteraceae, and Araceae, all of which are in the order Alismatales. The result shows that *C. rotundata* clusters in a clade with *Syringodium isoetifolium* (MZ325253.1), and this clade clusters with *Halodule uninervis* (OR098797.1). All three species are in the family Cymodoceaceae.

## Discussion and conclusion

The chloroplast genome is important in plant molecular evolution research due to several advantages. It is small and has already been extensively characterized at the molecular level, providing foundational information to support comparative evolutionary studies. Additionally, the rate of nucleotide substitution in the chloroplast genome is relatively slow and therefore provides the appropriate level of resolution for studying plant phylogeny and evolution (Clegg et al. 1994). This study is not only the first to sequence and characterize the complete chloroplast genome of *C. rotundata* but also the first for the genus Cymodocea. With this, the complete chloroplast genome of 3 out of 19 species in the family Cymodoceaceae has been sequenced so far, which is a low proportion (Ruan et al. 2021; Liu and Wu 2023). We suggest that more studies are needed to explore the genetic information of Cymodoceaceae. In the BI phylogenetic analysis, the results indicated that all the species in the family Cymodoceaceae clustered closely and that Cymodoceaceae shared a close relationship with the family Ruppiaceae first, then with the family Zosteraceae. Cymodoceaceae, Ruppiaceae, and Zosteraceae clustered in a clade, suggesting a common ancestor. This result was in accordance with the previous phylogenetic relationships of other seagrasses (Yu et al. 2020a; 2020c; Ruan et al. 2022), but with more species in Cymodoceaceae counted in. This study contributed to the organelle genomics research for the genus Cymodocea and the family Cymodoceaceae. Importantly, seagrass beds are declining globally (Short et al. 2011). According to NCBI data, the chloroplast genomes of 15 out of all 74 seagrass species have been sequenced. More organelle genomics data on seagrass need to be collected in the future.

## Supplementary Material

Supplemental Material

## Data Availability

The genome sequence data that support the findings of this study are openly available in GenBank of NCBI at [https://www.ncbi.nlm.nih.gov] under accession no. OQ735398. The associated Bio-Project, SRA, and Bio-Sample numbers are PRJNA963086, SRR24356035, and SAMN34435702, respectively.
