# Supplementary material for: The first complete chloroplast genome of Cymodocea rotundata Asch. & Schweinf. 1870 (Cymodoceaceae), an Indo-Pacific seagrass
Source: Mitochondrial DNA B Resour. 2024 Nov 26;9(11):1620–5. doi: 10.1080/23802359.2024.2432370 (PMC11610230; doi:10.1080/23802359.2024.2432370)
Supplement: Supplemental Material [file TMDN_A_2432370_SM2247.docx]

**
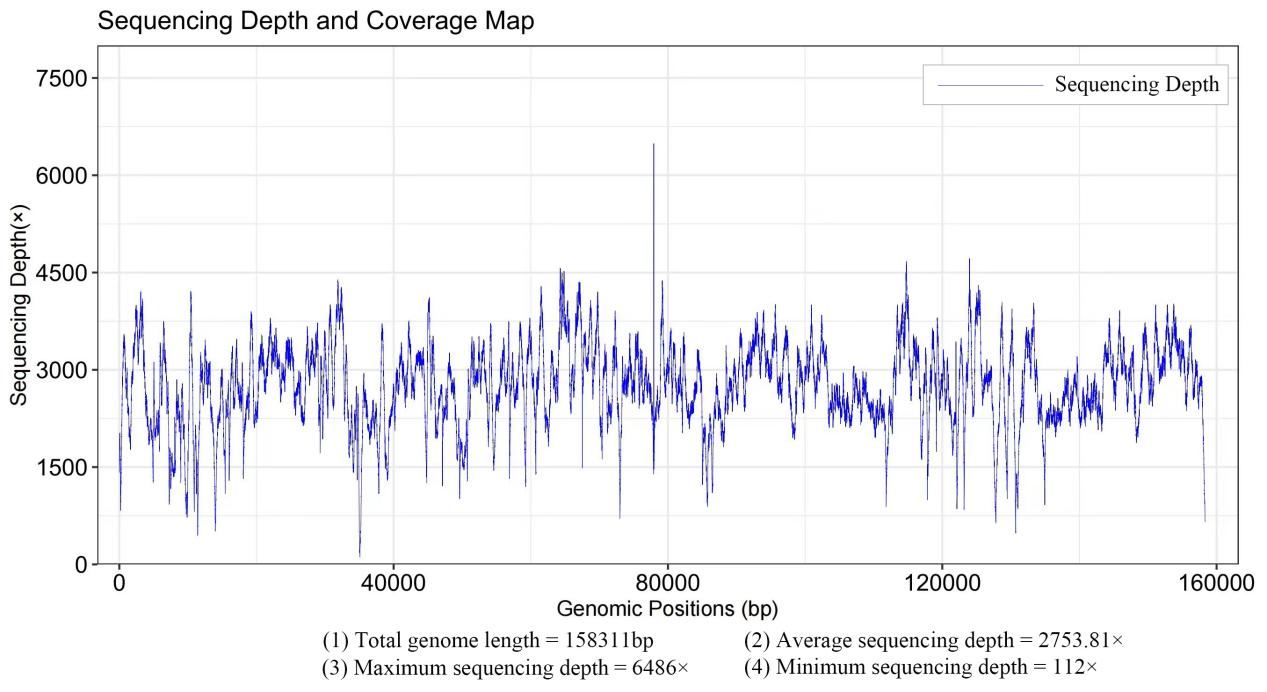
**

**Figure S1.** The map of sequencing depth, representing the sequencing depth on the complete chloroplast genome. The x-axis indicates the location of each base in the genome (in base pairs, bp) and the y-axis indicates the coverage depth (×). The blue line indicates the sequencing depth at each base of the genome.


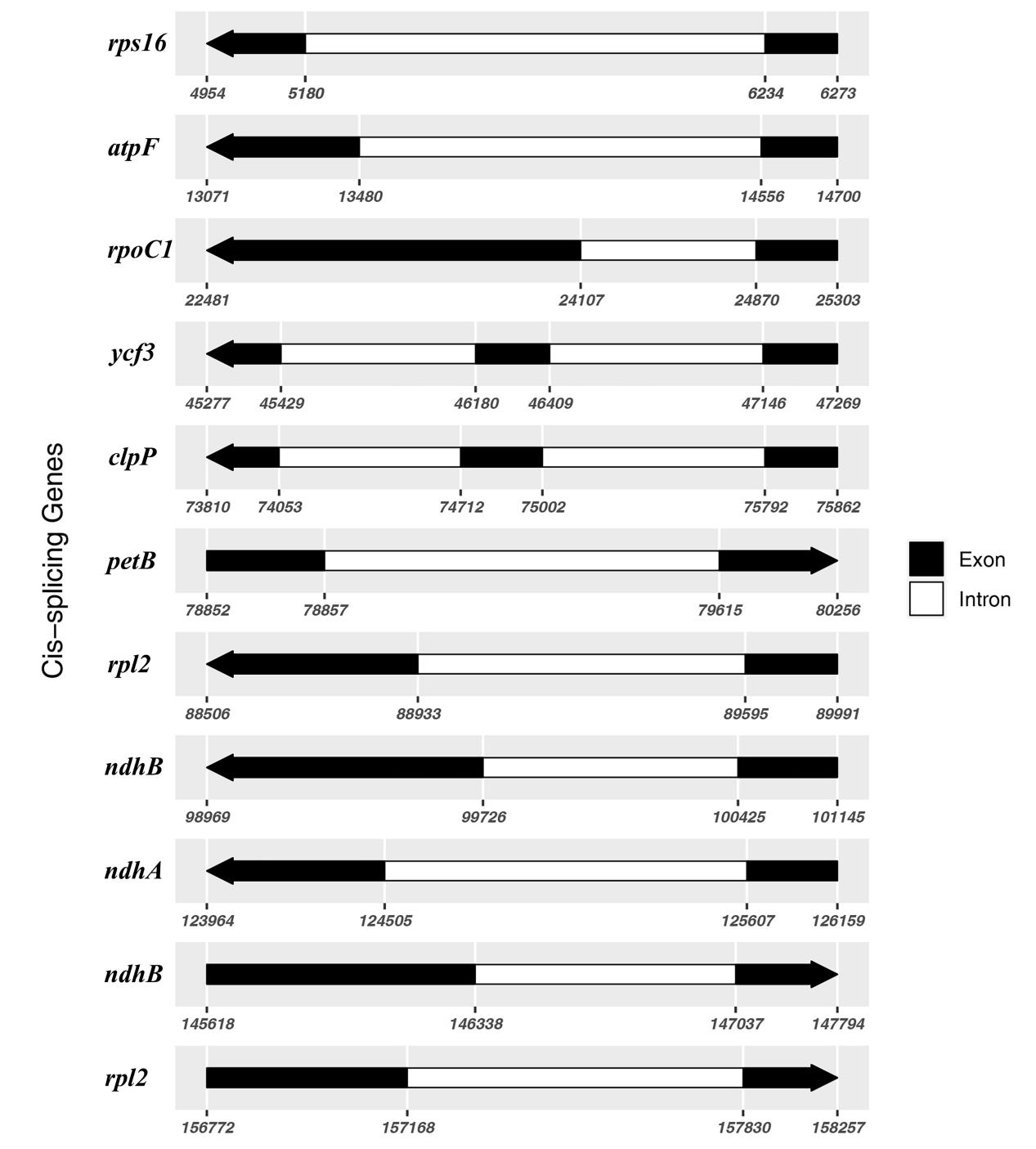


**Figure S2.** The map of the 11 cis-splicing genes (*rps16*, *atpF*, *rpoC1*, *ycf3*, *clpP*, *petB*, *rpl2*, *ndhB*, *ndhA*, *ndhB*, *rpl2*), including a duplicated *ndhB*. Nine of them have 1 intron and 2 exons, the other 2 have 2 introns and 3 exons. The genes are arranged from left to right based on their order on the chloroplast genome. The gene names are on the left top. The exons are shown in black and the introns are shown in white.

**
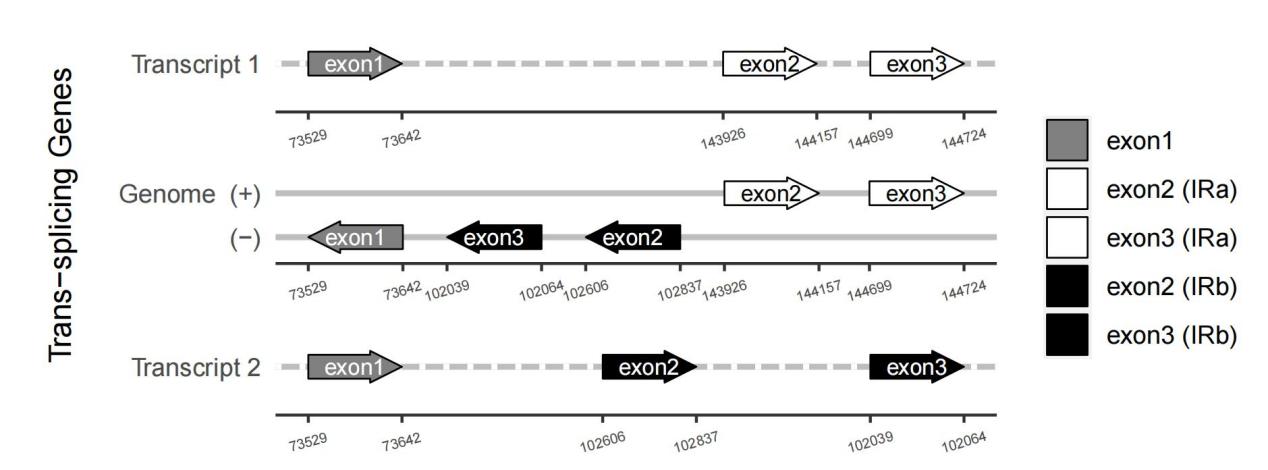
**

**Figure S3.** The map of the trans-splicing gene (*rps12*) on the chloroplast genome. The start and end positions on the pre-mRNA are shown below the line. The lines represent the genome plus (+) and minus (-)DNA strands. The arrowheads represent the corresponding exons of the rps12 genes.
